# Cellulose acetate based Complexation-NF membranes for the removal of Pb(II) from waste water

**DOI:** 10.1038/s41598-020-80384-0

**Published:** 2021-01-19

**Authors:** H. Idress, S. Z. J. Zaidi, A. Sabir, M. Shafiq, R. U. Khan, C. Harito, S. Hassan, F. C. Walsh

**Affiliations:** 1 Department of Polymer Engineering and Technology, University of the Punjab, Lahore, 54590 Pakistan; 2 Institute of Chemical Engineering and Technology, University of the Punjab, Lahore, Pakistan; 3 Electrochemical Engineering Laboratory, Faculty of Engineering and Environment, Engineering Sciences, University of Southampton, Highfield, Southampton, SO17 1BJ UK; 4 Industrial Engineering Department, Faculty of Engineering, Bina Nusantara University, Jakarta, 11480 Indonesia; 5 Mechanical Engineering, Faculty of Engineering and Physical Sciences, University of Southampton, Highfield, Southampton, SO17 1BJ UK

**Keywords:** Environmental chemistry, Environmental impact, Materials science, Nanoscience and technology

## Abstract

This study investigates the removal of Pb(II) using polymer matrix membranes, cellulose acetate/vinyl triethoxysilane modified graphene oxide and gum Arabic (GuA) membranes. These complexation-NF membranes were successfully synthesized via dissolution casting method for better transport phenomenon. The varied concentrations of GuA were induced in the polymer matrix membrane. The prepared membranes M-GuA2–M-GuA10 were characterized by Fourier transform infrared spectroscopy, scanning electron microscopy, transmission electron microscopy, atomic force microscope and bio-fouling studies. Thermal stability of the membranes was determined by thermogravimetric analysis under nitrogen atmosphere. Dead end nanofiltration was carried out to study the perm- selectivity of all the membranes under varied pressure and concentration of Pb(NO_3_)_2_. The complexation-NF membrane performances were significantly improved after the addition of GuA in the polymer matrix membrane system. M-GuA8 membrane showed optimum result of permeation flux 8.6 l m^−2^ h^−1^. Rejection of Pb(II) ions was observed to be around 97.6% at pH 9 for all the membranes due to electrostatic interaction between CA and Gum Arabic. Moreover, with the passage of time, the rate of adsorption was also increased up to 15.7 mg g^−1^ until steady state was attained. Gum Arabic modified CA membranes can open up new possibilities in enhancing the permeability, hydrophilicity and anti-fouling properties.

## Introduction

Environmental pollution particularly in water has become real human health issue, due to hasty development of industrialization and urbanization^1^. Water a “Vehicle of Nature” is an essential part of our life, micro and macromolecular functions are carried out in the presence of water^2^.

Numerous harmful overwhelming metals such as lead (Pb II), mercury (Hg II), zinc (Zn II), cadmium (Cd II) and arsenic (Ar II) are discharged from natural source of water^3–5^. These pollutants come from industrial reactions like manufacturing of batteries, electroplating and mining etc.^6,7^. Around the world researchers are working to overcome this environmental alarming situation of industrial wastewater pollution^8–12^.

Thus, the rejection of heavy metals from wastewater is important in order to prevent the environmental and human health. Pb(II) is the heavy metals that is commonly present in industrial wastewater and is indecomposable, which leads to many chronic diseases such as, anemia, nausea, convulsion, renal failure, cancer and indirectly disturb the metabolism of human beings^13–15^. To overcome these issues elimination of Pb(II) ions from waste water can be accomplished using conventional techniques. Ion exchange, chemical precipitation and electrochemical method, flocculation, photo-catalytic degradation, catalytic ozonation, coagulation these are not cost effective and exhibit incomplete removal of waste from water^16^. While adsorption methods are cost effective, they can be time consuming without external energy such as pressure^17,18^. To sum up, conventional processes have a drawback that they consume more time, energy and are costly in terms of operation and maintenance^16,19,20^.

Membrane technology is a clean, energy efficient, economical, and environmental responsive method for the selective separation of heavy metals^21–24^. Different types of membranes like Nano-filtration (NF), Reverse Osmosis (RO) and Ultra-filtration (UF) are used for removal of colloids, soluble organic particles, desalination and for heavy metals respectively^25^. NF is a pressure driven membrane process worked on Donnan exclusion and steric effect with improved transport phenomenon and most economical for the rejection of heavy metals from industrial wastewater^4,21,26–29^. The heavy metals in aqueous solution cannot be rejected by using simple Nanofiltration membrane whereas, complexation NF is well appropriate for the rejection of heavy metal.

Researchers are inspecting the formation of novel multifunctional nanomaterials-based membranes to development the sterilized systems and also reduce or reject inorganic and organic impurities from water. The adaptability of being able to add functional nanoparticles to the membrane, make it applicable for water purification^30^.

Recently different strategies have been examined for the development of effective and low cost biopolymers for wastewater treatment. Among these bio-based polymeric matrix membranes such as carboxymethyl cellulose, chitosan, sodium alginate and cellulose acetate (CA) are the promising one for increased hydrophilic character permeability and excellent selectivity towards heavy metals^15,31^. Bio-based polymers are renewable, abundant and economical, by chemical and physical means having capacity to join with different molecules^32–35^.

Furthermore, CA a bio-based polymer is used as a polymer matrix membrane due to better film forming ability, biocompatibility, eco-friendly, having important biomedical applications, potential affinity for water permeation and metal ion rejection properties^21,36–38^. However due to the poor mechanical and thermal properties CA alone is not suitable for NF performance^35,39,40^. To overcome this CA is incorporated with different materials like carbon nanotubes (CNTs), fume silica, ZnO nanorods, sepiolite, zeolite and graphene oxide (GO)^41,42^. Due to high mechanical as well as chemical properties and cost effectiveness the carbon-based graphene and GO used for membrane modification^43^.

GO is based on single layer of carbon atoms having size in nanometers. GO is a biocompatible, non-toxic, hydrophilic material with high surface area having good adsorption ability for divalent Pb II, which make it to be used for wastewater treatment. GO is bio adsorbent, thermally and mechanically stable inorganic material incorporated in membrane matrix that helpful in gas separation, pervaporation, dehydration, nanofiltration and wastewater treatment^44,45^. To increase the total bonding strength of organic and inorganic substrates organofunctional salines are extensively used as coupling agents. Bifunctional silanes are vinyl triethoxysilane (VTES), having vinyl group attached with silicon and hydrolyzable ethoxy group. The presence of the above mention groups increases chain elasticity and enhances bonding properties^46^. Modification of bio-polymer (CA) with VTES-GO showed good adsorption capability to reject the ions of heavy metal from industrial wastewater for better permeability flux and selectivity^44,47^.

Biofouling is a problem that causes microbial pathogenicity and hindrance in properties of material performance. Polymeric materials possessing antimicrobial properties like chitin, sodium alginate, gum arabic (GuA) etc. should be used to overcome the issue^48^**.** Arabic gum (GuA) is a biocompatible, biodegradable, and non-toxic polysaccharide that can be removed from the acacia tree, and has been used in membranes as a filler, possesses highly branched structure which comes from species of leguminous tree well adapted to Sudan and Sahelian agroecology of Africa^16^. It is hydrophilic natural polymer and nontoxic which can also be used for biofouling vindication and develop mass-transfer boundary layer on membrane surface^48^.

The present study focus on the performance of newly synthesized environmentally favorable polymer matrix (CA/GO) complexation-NF membranes tethered with GuA for Pb(II) rejection. These novel membranes are studied on dead end nanofiltration process to attain better performance with improved transfer phenomenon of membrane such as permeation flux and Pb(II) ion rejection. The functional group analysis, thermal stability, surface morphology, surface roughness and anti-bacterial properties of membranes were measured by Fourier transformed infrared spectroscopy, thermogravimetric analysis, scanning electron microscope, transmission electron microscope, atomic force microscope, and bio-fouling test respectively. Whereas similar membrane study for ion removal have been conducted deploying energy-dispersive X-ray spectroscopy^49,50^ and X-ray photoelectron spectroscopy^51,52^.

## Experimental

### Materials and methods

Analytical grade Cellulose Acetate (CA) with 39.0‒40.3% acetyl content obtained from DAE JUNG. Graphite from RIEDEL-DE HAEN GERMANY, H_2_O_2_ aqueous solution 35% obtained from RCI Lab scan Ltd. NaNO_3_ procured from PNEREAC QUIMICA SA, Hydrazine from BDH chemicals Ltd. All other analytical grade chemicals such as EG (≥ 99%), Dimethyl formamide (DMF solvent), Gum Arabic (GA), H_2_SO_4_ and KMNO_4_ were purchased from SIGMA-ALDRICH, USA. All the chemicals were used without further purification.

### Formation of graphene oxide (GO) by Hummer’s method

Graphite powder (2 g) and NaNO_3_ (2 g) were dissolved in 50 ml of H_2_SO_4_. Beaker was placed in an ice bath at continuous stirring for 2 h, maintaining a temperature at 5 °C. After that KMNO_4_ (12 g) was added in solution dropwise as an oxidizing agent, maintaining a temperature at 15 °C for 4 h. The solution was stirred continuously for 48 h until a brownish suspension was formed. Distilled water (100 ml) was added slowly into the beaker; reaction mixture was heated up to 98 °C for 1 h. Then 200 ml of distilled water was added again for further dilution while stirring was kept on. H_2_O_2_ (10 ml) was added dropwise in a solution, till appearance of yellow color which showed reaction termination. For purification purpose the mixture was rinsed out with distilled water (10×). The final product was vacuum dried at 25 °C, resulting in the formation of fine powdered GO.

### Modification of GO with vinyl triethoxysilane (VTES)

**Scheme 1:**
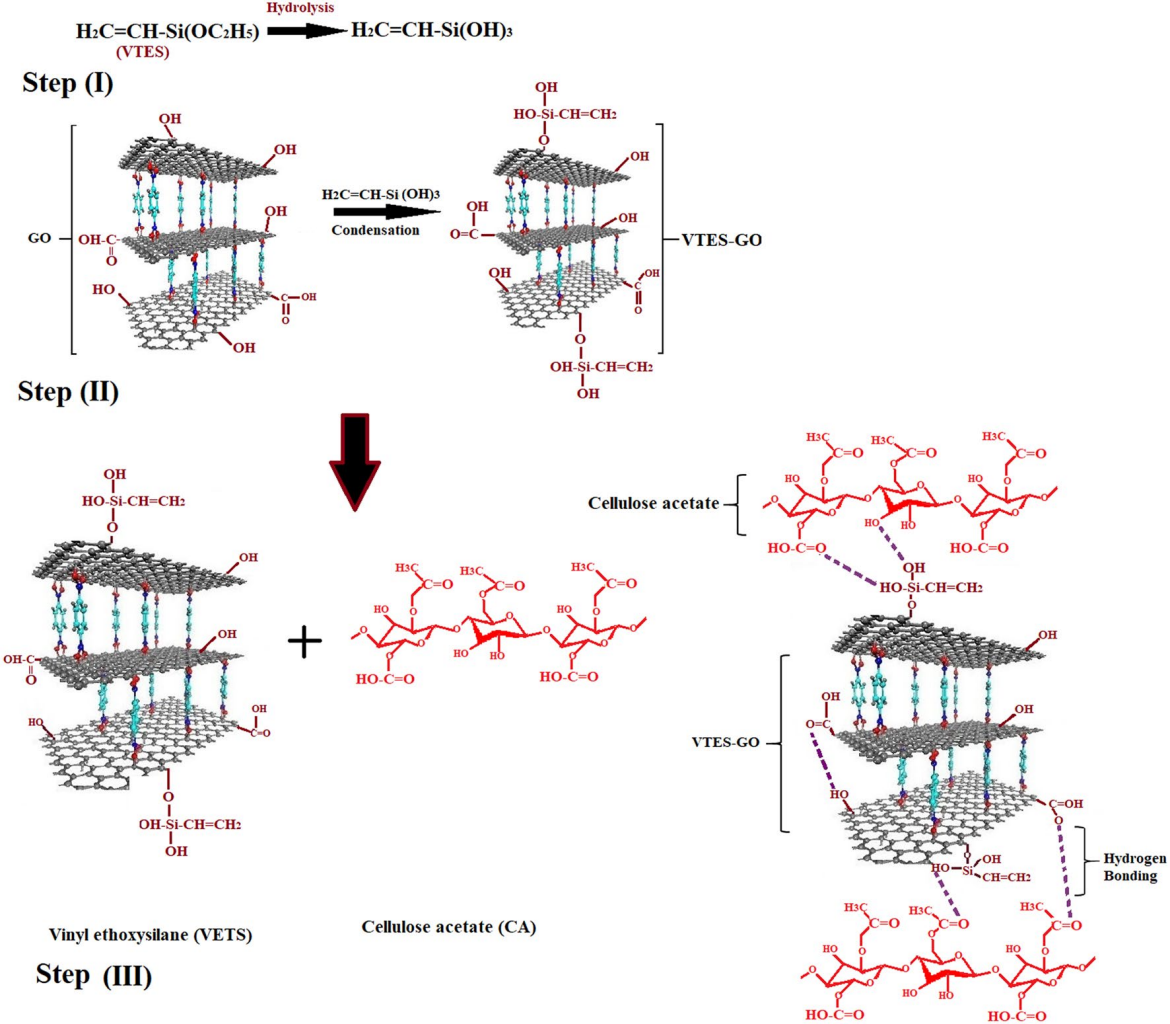
Step (I) Hydrolysis of vinyl triethoxysilane (VTES), Step (II) Modification of GO with VTES, Step (III) Structure of cellulose acetate modified with VTES-GO.

Indigenously synthesized GO was mixed with VTES (2 ml), HCl was taken about 0.5 v/v water in flask. To produce functionalized GO solution, hydrolysis of VTES and condensation of GO was carried out at 75 °C for 2 h. At the end of this reaction VTES modified GO was obtained filtered, and rinsed with deionized water for its neutralization. Then VTES modified GO was placed in vacuum oven at 60 °C until constant weight obtained. Scheme 1 Step (II) shows the modification of GO with VTES.

### Fabrication of cellulose acetate (CA)/VTES-graphene oxide (VTES-GO)

CA (1.5 g) was added in DMF and stirred continuously for 2 h, maintaining temperature at 60 °C. Then varying amount of VTES-GO (1–5 wt%) was added in CA homogenous solution. In Scheme 1 step (II) shows the structure of cellulose acetate modified with VTES-GO.

### Casting of membranes

The five sample mixtures of CA/VTES-GO (1–5 wt%) were casted in glass petri dishes carefully maintaining same thickness of each membrane. Then petri dishes were placed at 50 °C in vacuum oven for 24 h. These membranes were removed from petri dishes using doctor’s blade. Five final synthesized membranes were analyzed for lead rejection to bring forth the optimum filler CA/VTES-GO (3 wt%) was further named as M-GuA0, established optimal membrane for Pb(II) rejection (52%), then proceeded for further treatment.

### Fabrication of complexation NF network by incorporation of GuA

Gum Arabic (GuA) was added in different concentrations into M-GuA0 sample as shown in Table 1. Primarily six different solutions were stirred continuously for 2 h maintaining a temperature at 60 °C. These homogeneous solutions were casted as mentioned in “Casting of membranes” section.

**Table 1:** Concentration of Gum Arabic, Surface roughness (RMS) and Optical density in CA/VTES-GO polymer matrix.

Sample name	Gum Arabic (wt%)	Root mean square RMS (nm)	OD (600 nm)
M-GuA0	0	153	1.1133
M-GuA2	2	210	1.0603
M-GuA4	4	235	1.0030
M-GuA 6	6	310	0.9420
M-GuA8	8	361	0.8467
M-GuA10	10	220	0.667

## Characterization

### Fourier transform infrared spectroscopy (FTIR)

ATR-FTIR spectra of control M-GuA0 and modified membranes M-GuA (2–10) were recorded using FTIR (IR Prestige-21 Shimadzu, from Japan). The internal reflection was determined by zinc selenide (ZnSe) crystal. Frequency range of FTIR was 4000–400 cm^-1^ number of scans were 120 with resolution power of 4 cm^−1^ used for analysis.

### Thermogravimetric analysis (TGA)

The change in thermal stability of control M-GuA 0 and complexation –NF (M-GuA 2–10) the membranes as a function of time and temperature, using TGA. The results analyzed by instrument Mettler Toledo, TGA/SDTA851e under nitrogen flow (15 ml min^−1^). Analysis was carried out at 1200 °C.

### Scanning electron microscope (SEM)

Surface morphology and cross-sectional images of membranes (M-GuA0–MGuA10) were determined by SEM (S-3400N Hitachi, USA). To analyze the sample instrument was treated under very low vacuum. Denton Vacuum sputtering Automatico Desk IV were used for gold sputtering. Within the vacuum chamber sputtering will generate vapors, which in turns condensed in the form of thin film on to the sample.

### Transmission electron microscope (TEM)

Transmission electron microscopy (TEM) images of membrane samples were performed using a Philips CM 200 with EDS probe.

### Atomic force microscopy (AFM)

Topography and surface roughness of membranes M-GuA0–M-GuA10 were obtained by AFM (SPM9500J3, Shimadzu) without further preparation of samples. Sample having surface area of 5 × 5 µm was placed on holder. Cantilever tip was oscillated on the sample sinusoidally with 350 kHz resonating frequency, slightly in contact with the down stroke of each sample. The surface roughness of membrane was calculated by using Mean Roughness (Ra). It represents the mean value of surface relative to center of plan, and the plan enclosed the volume by the image above and below this plan are same. It is calculated by Eq. (1)

1 $$ {\text{Ra}} = \frac{1}{LxLy}\mathop \smallint \limits_{0}^{lx} \mathop \smallint \limits_{0}^{ly} \left| { f\left( {x,y} \right)} \right|dxdy $$

where f (x, y) is surface relative to the center of plane and Lx and Ly represent the dimensions of surface in x and y directions respectively. Therefore, root means square (RMS) values were obtained from AFM images, which in turn obtained from average of the values measured in random RMS values calculated by Eq. (2)

2 $$ RMS = \left[ { \left( \frac{1}{Ae} \right)\mathop \smallint \limits_{0}^{Lx} \mathop \smallint \limits_{0}^{Ly} Z^{2} \left( {x,y} \right)dxdy)} \right]^{1/2} $$

### NF membrane performance

Dead end filtration system (Sterlitech HP4750 Stirred Cell made of steel 316) was used for control and modified M-GuA0–M-GuA10 membrane for permeation flux and rejection of lead salt (PbNO_3_)_2_ on laboratory scale. Effective membrane surface area was 14.6 cm^2^. Pb(II) salt solutions of different concentrations (0–20 mg l^−1^) was taken at pressure of 1–6 bar for 15 h. Concentration gradient of selective ion transfer was developed at the interface of membrane due to concentration polarization. In order to get rid of concentration polymerization phenomena and precipitation of salt in NF, the solution was kept stirred at 800 rpm vigorously at pH of 1–10. Pb(NO_3_)_2_ was used to prepare stock solution and 0.01 M NaOH and HCl used to adjust the pH of solution. Membrane permeation flux was calculated by using Eq. (3)

3 $$ F = \frac{V}{t X A} $$

F = permeation flux in (l m^−2^ h^−1^), V = Volume of permeate unit in (l), T = time in (h), A = thin film area in (m^2^).

Salinity meter (TRACEABLE VWR, ISO 17025 Accredited) were used to determine the salt rejection of permeate.

### Contact angle

Contact angle measurements were carried out using a Goniometer (DIGI DROP, KSV Instruments). The sessile drop method was used to measure the contact angle of de ionized water on the dehydrated surface of the synthesized membranes. After the discharge of distilled water, on the membrane surface the image was captured and contact angle was measured. The given data were the mean of five contact angle values for each membrane sample.

### Bio-fouling properties

Bio-fouling test was prepared by using *Escherichia coli* according to JIS L 1902–2002 approach. Conical flasks having solution, were placed in autoclave at 121 °C for 15 min at 15 psi. 100 ml of DH5 alpha *E. coli* strain was injected into the flasks. Then membranes of different compositions M-GuA0 to M-GuA10 were added into it, flasks were put into incubator at 37 °C for 18 h. Optical density (OD) was measured by using a spectrometer at 600 nm.

## Results and discussion

### Fourier transform infrared spectroscopy

**Figure 1:**
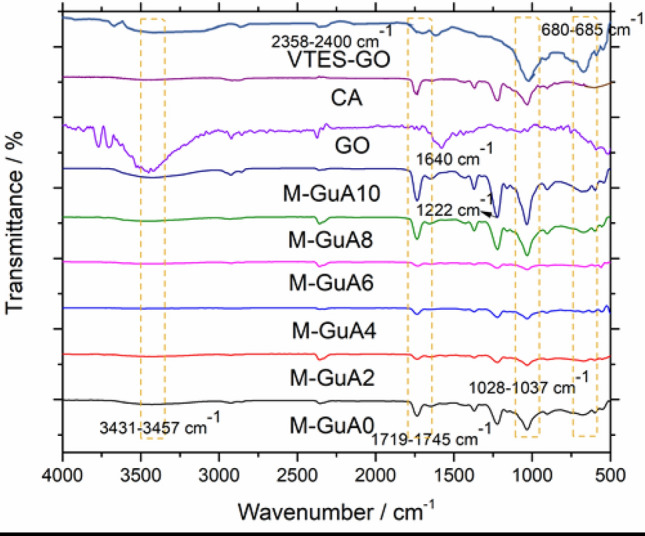
FTIR spectra of GO, CA, VTES-GO, and M-GuA membranes.

**Scheme 2:**
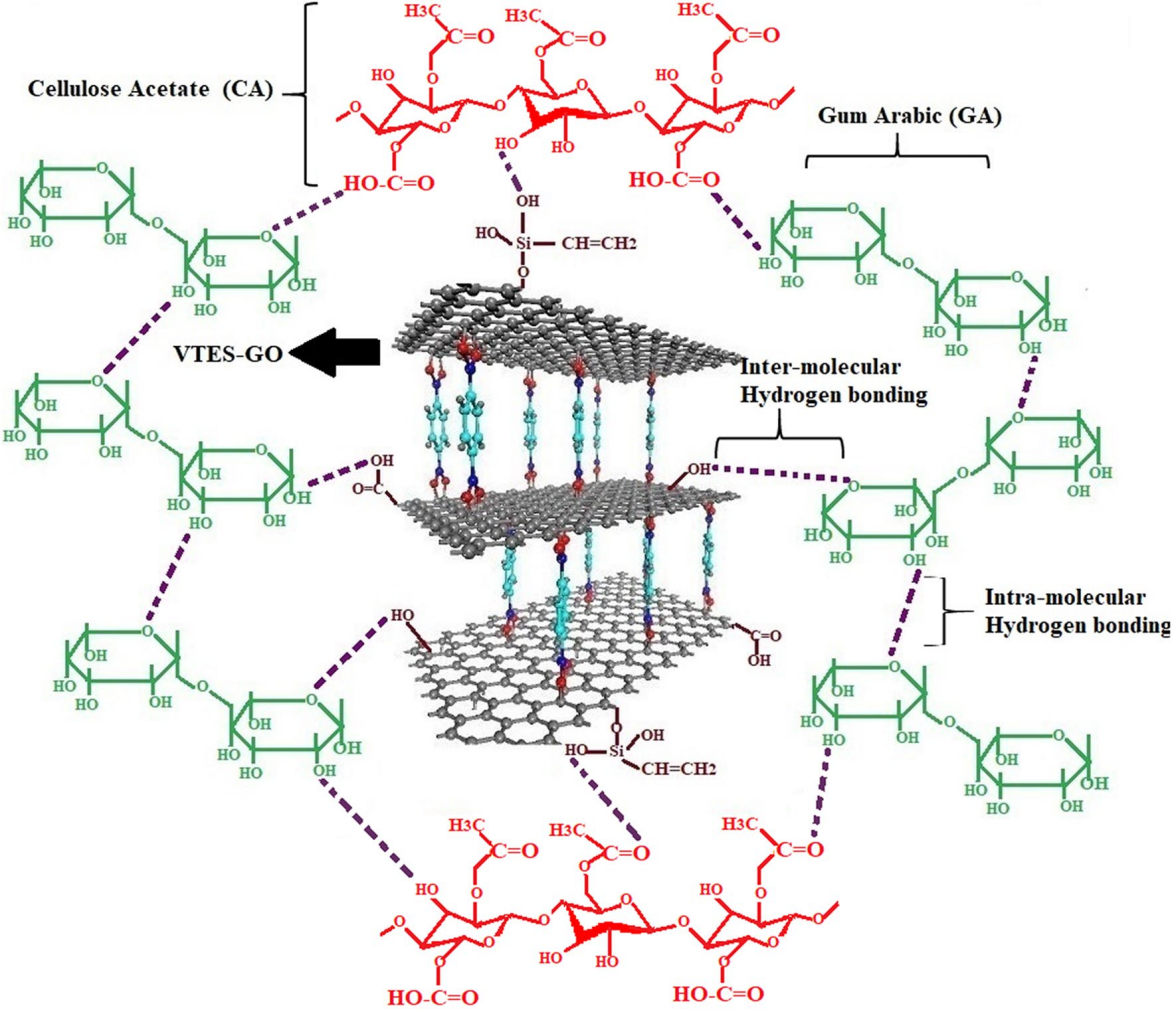
Complexation-NF network of Inter and intra molecular hydrogen bonding between CA, GO and GuA molecules.

FTIR spectrum of VTES-GO, M-GuA0 (control) and modified membranes from M-GuA2–MGuA10 are given in Fig. 1. FTIR was employed to confirm the interaction between CA, GO and GuA. The observed band at 3431–3482 cm^−1^ was associated with the ‒OH stretching vibrations due to inter-molecular intra-molecular hydrogen bonds increased as concentration of Gum Arabic increased shown in (Scheme 2)^40,53^. The band observed from 2358 to 2400 cm^−1^ were specific for C–C bending at low vibration^54^. The GO sample showed a strong characteristic peak at 3431–3482 cm^−1^ for ‒OH stretching and 1640 cm^−1^ for aromatic C=C^55^. The between 1719 and 1745 cm^−1^ confirmed the presence of C=O stretching band present in CA and VTES-GO^56,57,58^. The presence of band at 1037 and 1222 cm^−1^ were specific for C–O–C acyclic and cyclic groups of CA and GuA respectively^59^. While weak band at 908 and 1155 cm^−1^ confirmed the presence of pyranose ring and saccharide structure CA and GuA^40,60^ and 680–685 cm^−1^ were the Si–O stretching vibration peaks^61^.

### Thermogravimetric analysis (TGA)

**Figure 2:**
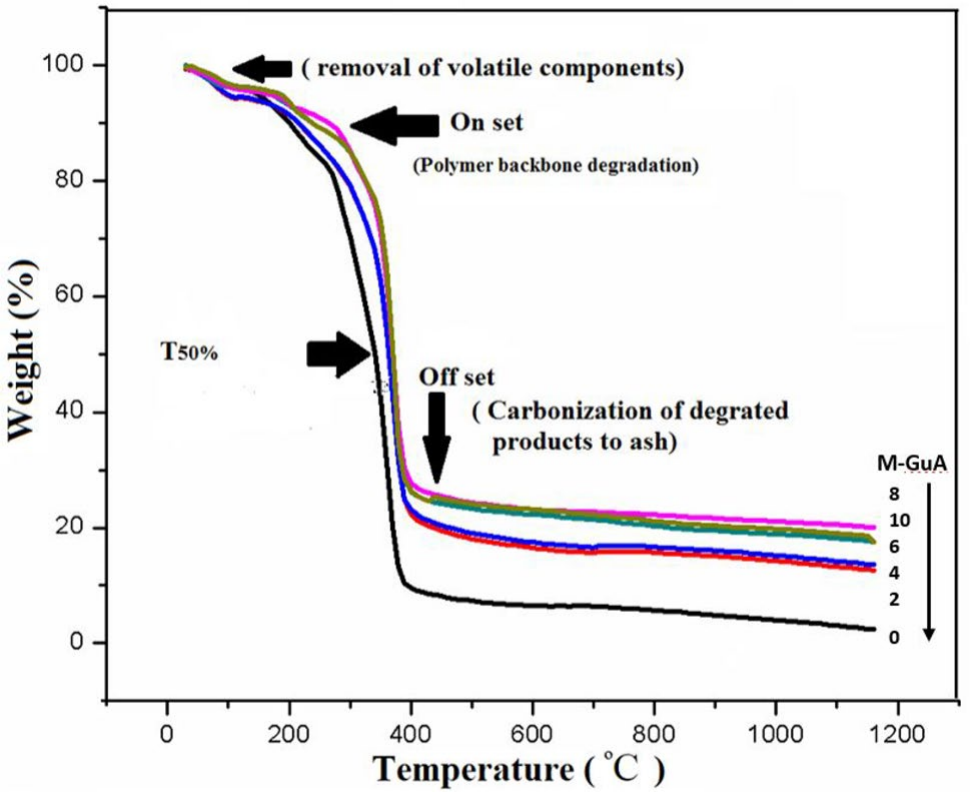
TGA of M-GuA membranes at various percentage weight loss. The number on the bottom right denoted the sample code, which are M-GuA8, 10, 6, 4, 2, 0, respectively from top to bottom.

Thermal degradation of control and modified membranes were studied in form of percentage weight loss given in Fig. 2. Three main steps were involved in thermal degradation analysis of polymer matrix membranes. First stage occurred between 30 and 250 °C, this degradation was due to removal of moisture contents and volatile matter from M-GuA0–M-GuA10. Second step involved onset temperature (T_onset_) started from 250 to 400 °C showing 70 wt% loss_._ The reason was the degradation polymer backbone and by deacetylation of CA^62,63^. Third step involved the offset temperature (T_offset_) started from 400 to 1160 °C, revealed carbonization of degraded products to ash because no change in mass occurred^64,65^. Table 2 revealed that degradation temperature of M-GuA8 was high as compare to control M-GuA0. The reason behind this was the homogenous dispersion of Gum Arabic in M-GuA8 as compared to control. M-GuA10 showed less degradation temperature, due to accumulation of Gum Arabic in the membrane which results in breakdown of main polymer chains at low temperature as compared to M-GuA8^27^. The weight loss at 50°C (T_50%_) of M-GuA0, M-GuA8 and M-GuA10 membranes were at 339, 370 and 369 °C, respectively. Which proved the M-GuA8 membrane was optimum and thermally stable membrane.

**Table 2:** TGA of M-GuA membranes at different % weight loss.

Sample code	T_onset_	T_50%_	T_offset_	Residue %
M-GuA0	300	339	387	10.31
M-GuA2	337	364	390	24.2
M-GuA4	338	365	393	24.3
M-GuA6	355	368	394	24.4
M-GuA8	356	370	396	30.2
M-GuA10	353	369	393	33.3

Temperatures are stated in °C.

### Scanning electron microscope (SEM)

**Figure 3:**
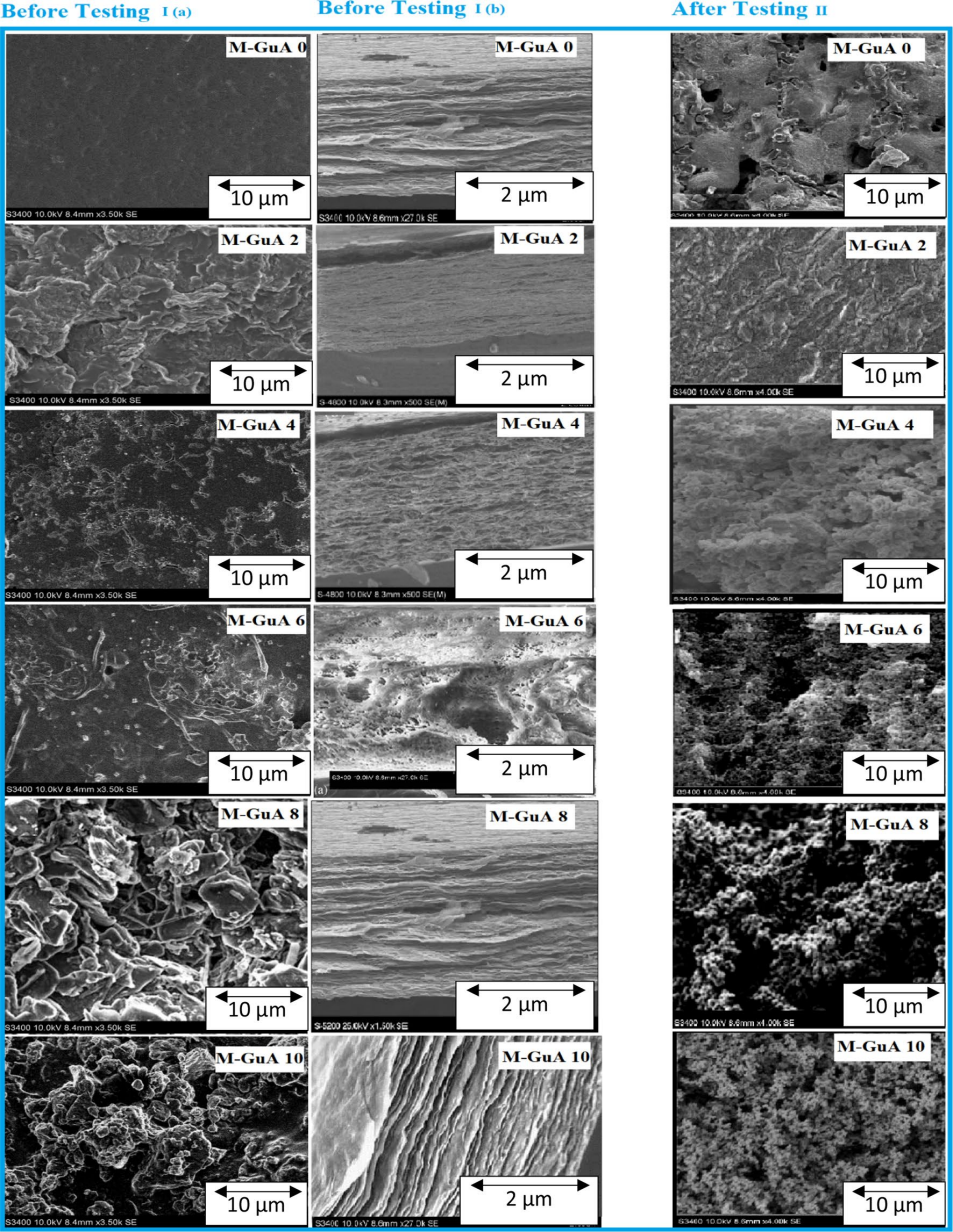
(I) Surface magnification (**a**) top surface (**b**) cross section of membranes at different magnifications (II) post SEM images of M-GuA membranes.

Figure 3 (I) shows the surface and cross-sectional morphology of (M-GuA0–M-GuA10) membranes using SEM. Surface morphological images label as (a) and cross-sectional images label as (b). M-GuA0 (a) showed tiny pores due to VTES having functionalized saline group imbedded on GO in CA polymer matrix, indicated strong adhesion between VTES-GO and CA^36^. M-GuA0 (b) showed that slight porous structure. M-GuA2 (a) membrane showed the clear dispersion of Gum Arabic, which increased porosity in membrane as compare to M-GuA0. Hydrophilicity or flux was improved as cleared in cross section of M-GuA2 (b). Compactness of membrane is lesser than control sample, chain opening phenomena occur. In (a) and (b) images of M-GuA4 and M-GuA6, membrane showed agglomeration of Gum Arabic, as compared to M-GuA0 and M-GuA2, which may lead to improvement in hydrophilicity and flux of membrane^66^. So, in case of M-GuA8, pore size was optimum as compared to M-GuA0 and M-GuA10. In case of M-GuA10 (a,b), moieties were formed which lead to smaller pores size in membrane structure proved that membrane become denser then M-GuA8. Presence of –OH groups in GuA, CA and VTES-GO results in enhancement of morphological structure^67^.

### Post SEM results

Figure 3 (II) shows the post SEM images of (M-GuA0–M-GuA10) membranes using SEM. Surface morphological images show the deposition of Pb(II) ions on the membranes surface. M-GuA0 (a) showed less deposition of Pb ions on the surface. M-GuA2 membrane showed the clear dispersion of Gum Arabic as compared to M-GuA0, so Pb(II) ions deposition was greater on it as compared to the control one. In images of M-GuA4 and M-GuA6 membrane showed much better accumulation of Pb(II) ions on surface, as compared to M-GuA0 and M-GuA2, maximum deposition of Pb(II) ions observed on M-GuA 8 membrane. In case of M-GuA10, the Pb(II) deposition was less than M-GuA 8, due to presence of GuA agglomeration in membrane.

### Transmission electron microscope (TEM)

**Figure 4:**
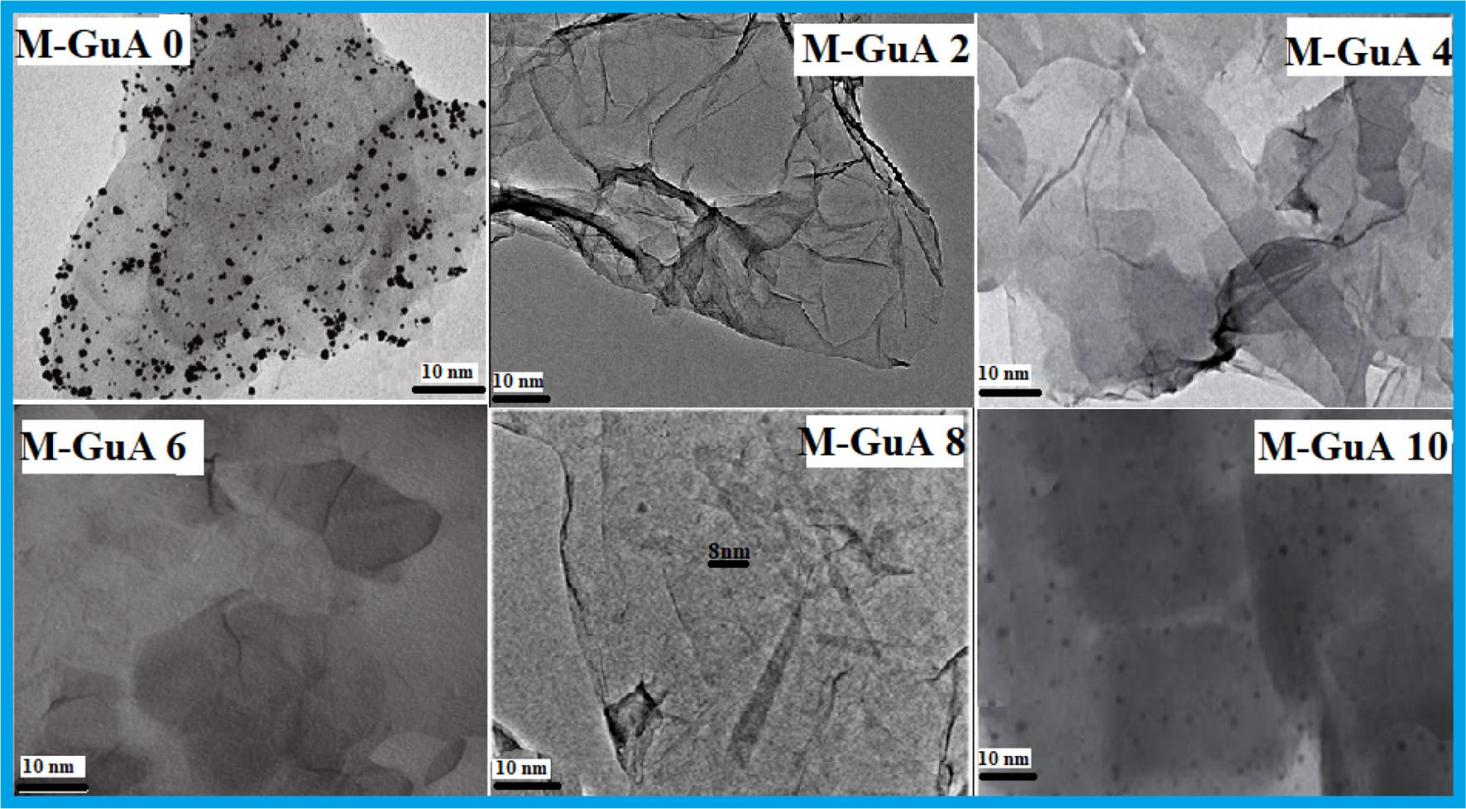
TEM images of M-GuA membranes.

Figure 4 shows the magnification of control M-GuA 0 and modified samples M-GuA10 at higher resolution of (10 nm). M-GuA0 showed the distribution of GO in matrix structure was dense as compared to modified samples. While in case of modified samples (M-GuA2–M-GuA8) wrinkles observed, because distribution of Gum Arabic nanoparticles was uniform as no free particles or aggregates were detected. In M-GuA8, size of nanoparticles found to be 8 nm as shown in Fig. 4. In case of M-GuA10 mottled structure observed which showed aggregation of Gum Arabic in it justified the absence of proper bonding between CA, VTES-GO and Gum Arabic^68^.

### Atomic force microscope (AFM)

**Figure 5:**
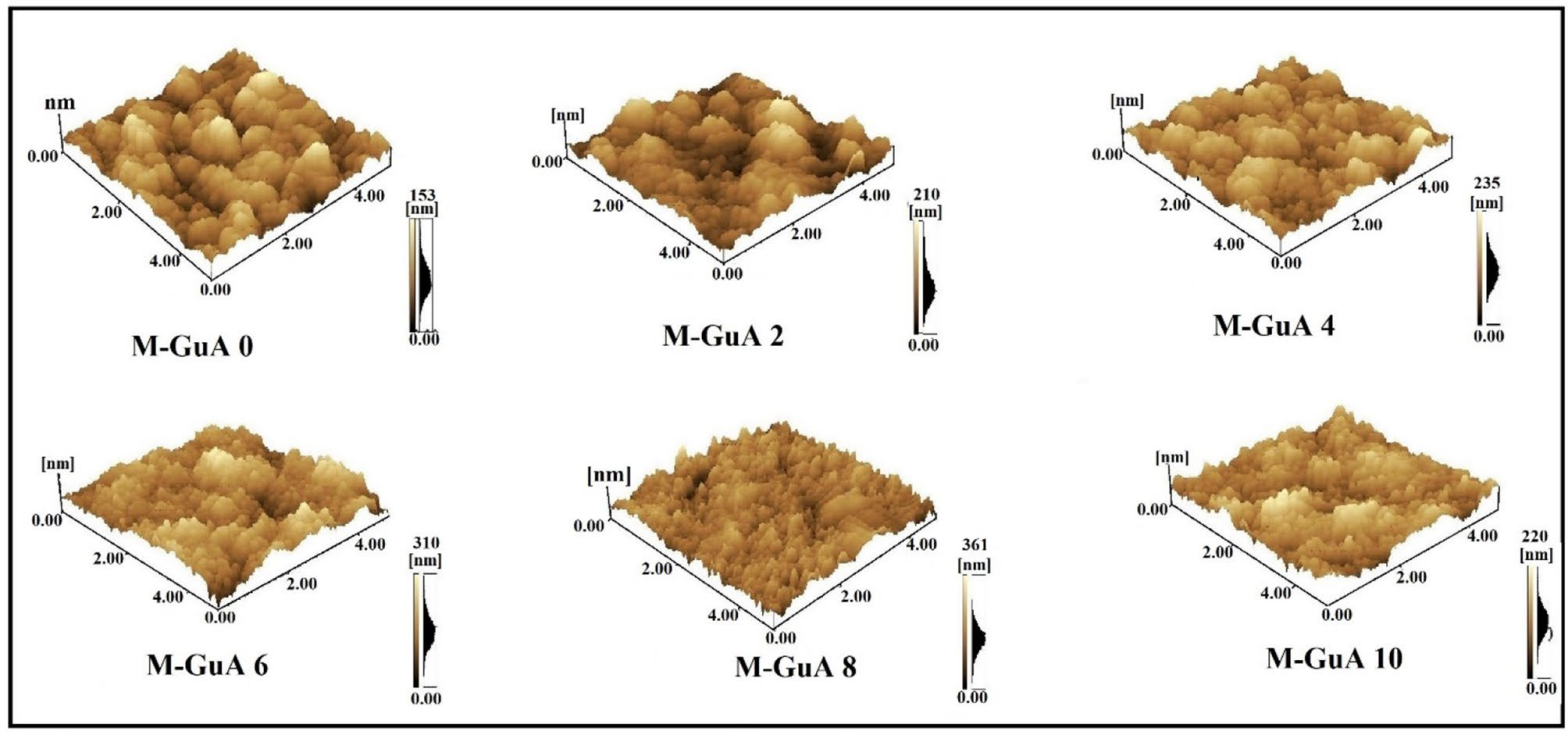
AFM 3-Dimentional images of M-GuA membranes.

Figure 5 shows the Surface topography and roughness of the membranes. The root mean square (RMS) value of polymer matrix membranes increased as concentration of Gum Arabic was increased from M-GuA0–MGuA8. M-GuA0 showed 153 nm less than all other samples as shown in Table 1. The reason behind is the absence of GuA in it, which causes less valleys and ridges compared to other membrane. M-GuA2–MGuA6 the surface roughness values continuously increased from 210 to 310 nm, indicated that addition of GuA increased the surface roughness. Increment in valleys and ridges means increment in roughness parameter and adsorption^69,70^. RMS value of M-GuA8 was 361 nm which was optimum one showed better adsorption as compare to control membrane. The addition of Gum Arabic increased the hydrophilicity and surface adsorption in the membranes^70^. Sudden decrease in RMS value 220 nm of M-GuA10 due to greater accumulation of filler in vicinity of membrane, which will ultimately cause decrease in flux and adsorption rate^71^.

### NF membrane performance

#### Effect of concentration of Pb(II) on permeation flux

**Figure 6:**
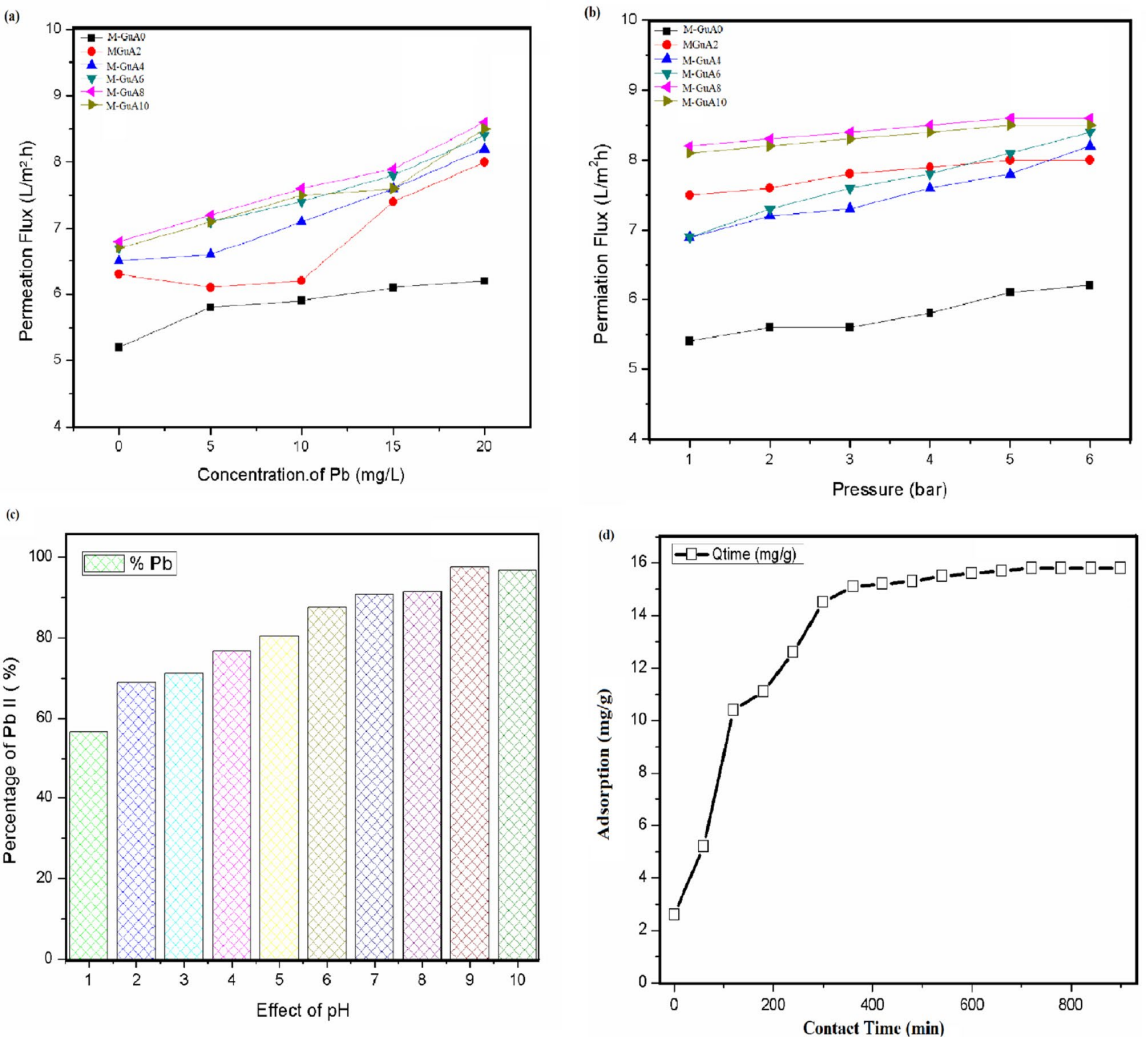
Effect of (**a**) concentration and (**b**) pressure on permeation of Pb(II), (**c**) Effect of pH on Pb(II) rejection, (**d**) Effect of contact time on adsorption of Pb(II).

The plot against concentration and flux of M-GuA membranes is given in Fig. 6a. Concentration of Pb(II) varied from 0 to 20 mg l^−1^. M-GuA0 showed less increase in permeation flux from 5.2 to 6.2 L m^−2^ h^−1^compared to M-GuA2. The optimum values observed in case of M-GuA8 membrane is 6.8–8.6 l m^−2^ h^−1^ then permeation flux decreased in case of M-GuA10. The reason behind is the compaction phenomena in which first permeation flux decreased and then increased. M-GuA8 show maximum flux rate (8.6 l m^−2^ h^−1^) at 20 mg l^−1^ of Pb(II). It indicated that the increase in concentration of filler in the polymer increased the flux of the membranes. Decline in values of M-GuA10 is due to greater concentration of Pb(II) ions in the vicinity of membrane^70^. High permeability leads to perfection in hydrophilicity of the membrane^67^. Other reason to explain this property of membrane is presence of OH^-^ group in CA, VTES-GO and Gum Arabic developed inter and intra-molecular hydrogen bonding in the membrane. Permeability raised up to a certain limit and then reduced when concentration of filler exceeded, because compactness occur in structure of membrane^67^.

#### Effect of operating pressure on permeation flux

Figure 6b shows effect of operating pressure on permeation flux. In Sect. 4.7.1, it was clarified that 20 mg l^−1^ conc. of Pb(II) gave maximum flux results, so it was considered as standard for pressure application. In control (M-GuA) membrane flux increased from 5.4 to 6.2 l m^−2^ h^−1^ but less than modified membranes from M-GuA2–M-GuA6 due to applied change in osmotic pressure.

Optimum values of permeation flux were observed in case of M-GuA8 are 8.2–8.6 l m^−2^ h^−1^ Increased permeation flux in case of M-GuA8 membrane was due to increased pressure, convective transport and polarization factor^72,73^. Whereas, the sudden decline in values of permeation flux in M-GuA10 from 8.1 to 8.5 l m^−2^ h^−1^ was due to increased change in osmotic pressure. Flux rate of salt (PbNO_3_)_2_ depend up on applied pressure, and water gradient^74,75^. This function leads to better rejection of metal from Pb(II) solution.

#### Effect of pH on Pb(II) % rejection

Figure 6c shows the effect of change in pH (1–10) on Pb(II) % rejection. Sudden reduction was observed in case of pH 10. Whereas, at pH 1 Pb(II) % rejection was 56.7%. Optimum rejection (97.6%) was observed for M-GuA8 at pH 9. It was cleared from results that low (pH 1–6) was not obligatory for better rejection^74^. Furthermore, charge present on membrane surface is effected by pH and in turn influence the metal ion^76^. VTES-GO behave as absorbent in membrane, the adsorption capacity of metallic species was enhanced with pH. It was also observed that from pH (1–9) the absorption capacity of membrane reduced in acidic medium compared to basic medium. The reason was, functional groups –COOH and –OH present on VTES-GO, CA and Gum Arabic respectively, deprotonated in acidic medium^6^. Another fact was lower pH also leads to neutralization of functional group due to which absorption power of cation Pb(II) decreased on membranes. Furthermore, struggle between H_3_O^+^ and metal ions leads to low adsorption^6,77^. In basic medium (pH 7–9), maximum Pb(II) rejection occurred due to conversion of Pb(NO_3_)_2_ to metal hydroxide. Electrostatic interaction is the reason when functional groups changes from -COOH and -OH to (-COO^-^ and -O^-^), which results high Pb(II) rejection. Moreover, at pH 10 rejection sudden decrease due to greater precipitation of metal hydroxides in solution except absorption^77^.

#### Effect of contact time on membrane performance

Effect of contact time was observed on optimum Complexation-NF membrane (M-GuA8) for 15 h as shown in Fig. 6d. It was well-defined from figure that adsorption rate at zero time is 2.6 mg g^−1^. Initially the value increased to 5.2 mg g^−1^ whereas, after 11 h values started continuously increasing from 10.4 to 15.7 mg g^−11^ till steady state was attained.

The reason acclaimed the adsorption related to the hydrated radii of Pb(II). Hydrated radii of Pb(II) is 94 pm, smaller hydrated radii indicates that absorption of Pb(II) greater on the surface of membranes^78^. Greater absorption was observed in first 11 h due to larger pores in membrane structure, which leads to fast transport of Pb(II) toward membrane surface^77^. VTES-GO behave as absorptive nanomaterial in membrane structure^6^.

### Contact angle

**Figure 7:**
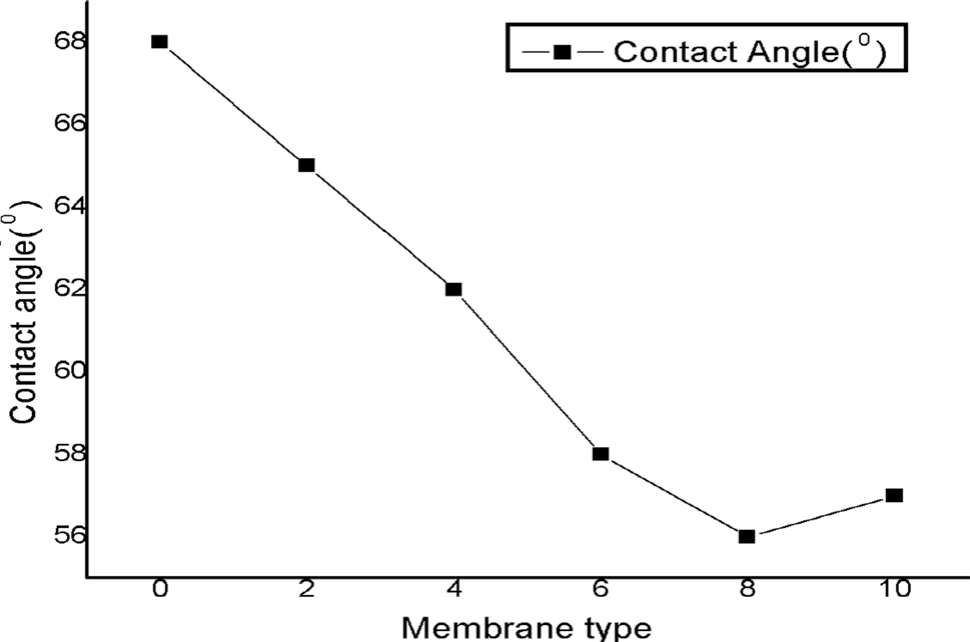
Shows the surface contact angle.

Contact angle of pure M-GuA0 (control) and modified membranes M-GuA2–M-GuA10 were analyzed to evaluate the surface hydrophilicity of the membrane are showed in Fig. 7, pure membrane (MGuA0) showed a larger value (68°) though modified membranes showed low values. The increase in Gum Arabic concentration illustrated decreased in contact angle from M-GuA2-MGuA 8. The membrane with lower contact angle would have higher hydrophilicity minimum value showed by M-GuA (56°) as the Gum Arabic decrease the contact angle, therefore increase the hydrophilicity^79,80^. In case of MGuA10 the contact angle value (57°) again increases because membrane become denser as discussed in “Scanning electron microscope (SEM)” section^66,67^. The contact angle of the averaged value for each membrane was presented in Fig. 7.

### Bio-fouling

**Figure 8:**
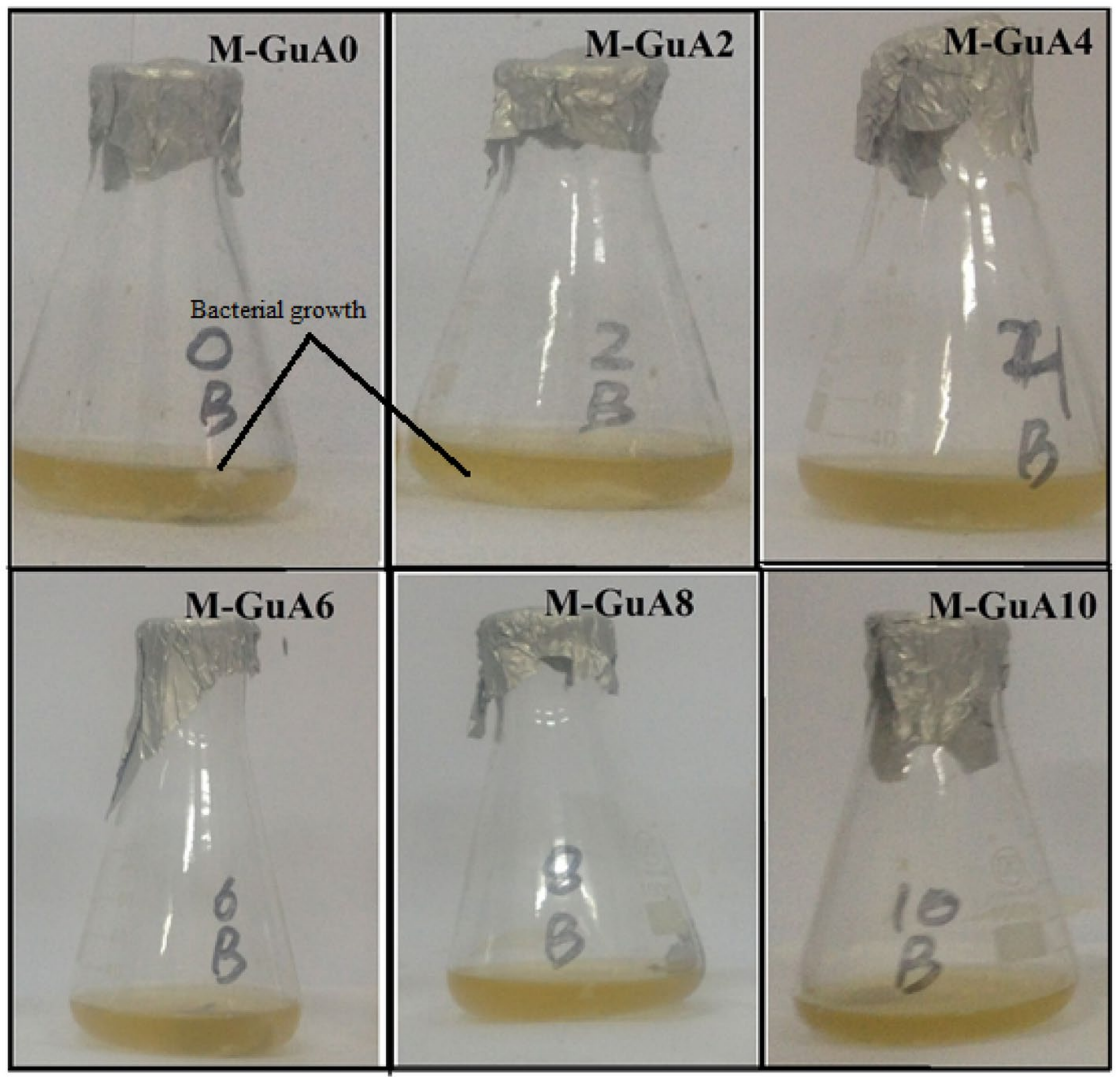
Bio-fouling of M-GuA membranes.

Figure 8 shows the bio-fouling images of M-GuA (0–10) membranes. The optical density (OD) of complexation-NF membranes M-GuA0–M GuA10 showed OD value from 1.0030 to 0.667. Control membrane (M-GuA0) showed cloudy image because of bacterial growth. OD value of M-GuA2, M-GuA4 and M-GuA6 membranes decreased gradually from 1.0603 to 0.9420, and having transparent images compared to control membrane. The reason was the addition of Gum Arabic in these membranes. The clear and transparent solution of M-GuA8 and M-GuA10 showed OD 0.8467 and 0.667 respectively. Addition of Gum Arabic led to less bacterial growth, because cells of *E. coli* died and they lost their cellular identity^67^. And Minor bacterial growth was due to repulsion between GuA and *E. coli*, since both *E. coli* and GuA having negatively charged surface^67,81^. Besides M-GuA membrane, one step synthesis method of functionalized GO can be deployed for nanofiltration membrane for the future work^82^.

## Conclusion

CA based membranes modified with VTES-GO/GuA as a filler enhanced the properties of permeation flux and Pb(II) ion rejection. The effect of pH, contact time, permeation flux and pressure on Pb(II) ion rejection has been studied using NF-membranes. It showed that optimum rejection was 97.6% at pH 9, which also revealed that low pH from 1 to 6 was not obligatory for salt rejection. The permeation flux due to varied pressure and concentration was 8.6 l m^−2^ h^−1^. Moreover, the functional groups were confirmed by FTIR, surface morphology observed by SEM and topographical images by AFM were justified the even distribution of GuA in M-GuA8. TGA results was revealed that stability depend on uniform dispersion of GuA in membrane. Bio-fouling results further confirmed that greater concentration of GuA enhanced bacterial rejection properties. The described results are due to unique properties of nanomaterial-based membrane and their convergence with current treatment technique present great opportunities to revolutionize water and waste water treatment, and improve the performance understanding of NF-membrane and can be important parameter for the fabrication of membrane on commercial scale.
